# Water Intake and Hydration Indices in Healthy European Adults: The European Hydration Research Study (EHRS)

**DOI:** 10.3390/nu8040204

**Published:** 2016-04-06

**Authors:** Olga Malisova, Adelais Athanasatou, Alex Pepa, Marlien Husemann, Kirsten Domnik, Hans Braun, Ricardo Mora-Rodriguez, Juan F. Ortega, Valentin E. Fernandez-Elias, Maria Kapsokefalou

**Affiliations:** 1 Unit of Human Nutrition, Department of Food Science and Human Nutrition, Agricultural University of Athens, 75 Iera Odos Str., Athens 11855, Greece; olgamalisova@yahoo.gr (O.M.); dathanasatou@gmail.com (A.A.); alekspepa@gmail.com (A.P.); 2 Institute of Biochemistry, German Sport University, Cologne 50993, Germany; m.husemann@biochem.dshs-koeln.de (M.H.); kirsten.domnik@gmx.de (K.D.); h.braun@dshs-koeln.de (H.B.); 3 Exercise Physiology Lab at Toledo, University of Castilla-la Mancha, Toledo 13071, Spain; ricardo.mora@uclm.es (R.M.-R.); juanfernando.ortega@uclm.es (J.F.O.); valentin.fernandez@uclm.es (V.E.F.-E.)

**Keywords:** hydration status, water intake, hydration indices, urine, blood, seasonality, country

## Abstract

Hydration status is linked with health, wellness, and performance. We evaluated hydration status, water intake, and urine output for seven consecutive days in healthy adults. Volunteers living in Spain, Germany, or Greece (*n* = 573, 39 ± 12 years (51.1% males), 25.0 ± 4.6 kg/m^2^ BMI) participated in an eight-day study protocol. Total water intake was estimated from seven-day food and drink diaries. Hydration status was measured in urine samples collected over 24 h for seven days and in blood samples collected in fasting state on the mornings of days 1 and 8. Total daily water intake was 2.75 ± 1.01 L, water from beverages 2.10 ± 0.91 L, water from foods 0.66 ± 0.29 L. Urine parameters were: 24 h volume 1.65 ± 0.70 L, 24 h osmolality 631 ± 221 mOsmol/kg Η_2_Ο, 24 h specific gravity 1.017 ± 0.005, 24 h excretion of sodium 166.9 ± 54.7 mEq, 24 h excretion of potassium 72.4 ± 24.6 mEq, color chart 4.2 ± 1.4. Predictors for urine osmolality were age, country, gender, and BMI. Blood indices were: haemoglobin concentration 14.7 ± 1.7 g/dL, hematocrit 43% ± 4% and serum osmolality 294 ± 9 mOsmol/kg Η_2_Ο. Daily water intake was higher in summer (2.8 ± 1.02 L) than in winter (2.6 ± 0.98 L) (*p* = 0.019). Water intake was associated negatively with urine specific gravity, urine color, and urine sodium and potassium concentrations (*p *< 0.01). Applying urine osmolality cut-offs, approximately 60% of participants were euhydrated and 20% hyperhydrated or dehydrated. Most participants were euhydrated, but a substantial number of people (40%) deviated from a normal hydration level.

## 1. Introduction

The evaluation of hydration status in the general population in free-living and/or under special conditions such as in disease or in the work environment is of unequivocal importance for public health. This is because dehydration is linked with reduced physical and cognitive performance [1] or disease [2,3].

Hydration status reflects the balance between water intake and loss. Water intake includes, approximately, 20% contribution of water from solid foods and 80% contribution of water from beverages and drinking water [4,5,6]. It follows that water intake, although mostly driven by thirst, depends on a variety of factors such as eating and drinking habits and preferences or availability of foods and beverages [7,8,9]. Water loss consists mainly from excretion of water in urine, respiratory water, feces and sweat [10]. Since the contribution of sweat in water loss is higher in a physically active person and in hot weather [11], water loss is affected by physical activity levels and season. Therefore, water loss is highly variable, even in healthy individuals, depending on the lifestyle of the individual and on environmental conditions or geographical location.

Data on water intake in relation to hydration status in population groups in free-living conditions are scarce. This constitutes a knowledge gap and consequently an obstacle in supporting initiatives for improving the hydration of the population. There is an urgent need to build databases on the estimation of water intake and of hydration status in the population.

Selecting the appropriate research tools for evaluating water intake and hydration status is crucial. Seven day diaries, in which all foods and beverages consumed are recorded, may present advantages in reflecting intake [12] compared with other tools, such as 24 h recall or food frequency questionnaires [13]. A synthesis of indices in urine and blood samples [3,12,14,15,16,17] is necessary for the evaluation of hydration status of individuals or population groups, as there is no single index to reflect hydration status [12,18,19]. Yet, measuring a series of hydration indices in samples collected over seven days instead of spot urine [20] may provide advantages since this approach incorporates factors that fluctuate during the week and affect hydration status, such as eating and drinking habits, physical activity, and environmental conditions.

The objectives of the study were to assess hydration status, water intake and urine output in summer and winter over seven days in a sample of healthy adults in three European countries.

## 2. Materials and Methods

Participating centers were the Agricultural University of Athens, Greece (GR), the German Sport University, Cologne (GER), Germany, and the University of Castilla La Mancha, Spain (ESP). The study was conducted in population living in the metropolitan areas of Athens, Cologne, and Toledo, respectively, in parallel and following identical protocols during winter (1–3/2013, 12/2013, 1–2/2014) and summer (6–8/2013, 6–7/2014). Five hundred and seventy three subjects aged 20–60 (39 ± 12 years) (51.1% males) with a BMI 25.5 ± 4.2 kg/m^2^ for males and 24.5 ± 4.9 kg/m^2^ for females were enrolled in the study. Subjects were adults aged 20–60 years with approximately equal numbers in each decade of life. Demographic factors such as ethnic origin, living conditions, marital status, and other were not considered to further stratify the sample.

The study protocol was approved by the Research Ethics Committee in each center involved (197/27-02-2012 for Agricultural University of Athens, Greece, 4/02/213-18 for University of Castilla La Mancha, Spain, 1/26-11-2012 for German Sport University, German). Written informed consent was obtained from all subjects. Exclusion criteria were disease (diabetes insipidus, renal disease, liver disease, gastrointestinal diseases or problems, cardiac or pulmonary diseases, disease that limits mobility including muscle-skeletal diseases, or orthopedic problems), pregnancy, lactation, hypertensive under severe salt restriction, taking drugs that are, or contain, diuretics, phenytoin, lithium, demeclocycline, or amphotericin, and following a high-protein and/or hypocaloric diet. Subjects were rescheduled or omitted if they caught flu (cold) or had fever, vomiting, and/or diarrhea or menstruation during the data collection period. Data from subjects who lost or gained more than 2% of body mass between day 1 and 8 were discarded. Additionally, data from subjects with values of creatinine excretion rate (CER) >3500 mg/day or <350 mg/day were revealing inaccurate 24 h urine collection [21], consequently four subjects who had CER >3500 mg/day were excluded from the analyses. Twenty-eight subjects did not complete the protocol (13 in winter, 15 in summer) for personal reasons.

### 2.1. Recruitment

Recruitment started two weeks before, and continued throughout, the study period. Recruitment strategy included invitations (a) sent by email to the non-academic and academic personnel of the three study centers; (b) uploaded on social media and published in local newspapers; (c) uploaded on internet sites related to nutrition; (d) distributed in paper at various non-academic places; (e) sent by email to other academic and social work institutions in the greater area of the centers involved (f) distributed at any seminar that the research teams were giving. Volunteers expressing interest for participating to the study completed a screen questionnaire in order to detect any of the exclusion criteria. If admitted to the study, subjects received the study protocol in writing, verbal responses to any questions they had on the purpose of the study, detailed instructions on study procedures including recording of food, drink and urination, and signed an informed consent form.

### 2.2. Study Protocol

Subjects entering the study received a small back pack containing instruction sheet for study protocol; a diary for recording urine volume; a kitchen scale readable to 1 g; a urine collection container; eight Zip-loc bags, seven of them containing 10 screw cap tubes (10 mL) for urine sampling, each labeled with subject code number, day, and urination time and one Zip-loc bag containing one screw cap tube (10 mL) for urine sampling in the morning of the eighth day. Additionally, subjects received a styrofoam box (30 × 50 × 20 cm) and/or ice packs for the storage of samples. Furthermore, each subject received (a) a seven day diary (7DD) to report in detail foods, drinks, water, wake up time, bed time; (b) a physical activity questionnaire (Short version of the International Physical Activity Questionnaires; IPAQ) [22] for each day of the week; and (c) a questionnaire including a series of questions regarding the profile of the individual, behavior and knowledge about hydration. A mini interview on motives and barriers to good hydration was conducted on the first day of the study period of each individual.

Subjects entered the study on different days of the week in order to achieve a reasonable distribution of starting days over the week. On study day 1 subjects arrived fasted at the study center between 7:00 and 9:30, bringing a weighted sample of their first morning urine void. Upon arrival, participants’ body height was measured was with mechanical sliding scale (Seca 711 Mechanical Sliding Weight Beam Scale) and mass measured with electronic digital scale (TANITA, Body Composition Analyser, TBF 300) wearing underwear and no shoes. They were also instructed to sit for approximately 15–20 min while filling in study questionnaires. Subsequently, a blood sample (5 mL) from a vein in the forearm was collected without stasis.

On days 1–7, while going about their normal daily routine, subjects recorded their food and drink consumption based on portion sizes and/or package information, collected and recorded the weight of each urination and of time of collection and retained a sample in a numbered tube, as instructed. Subjects stored the urine tubes in their refrigerator or in the styrofoam box using ice packs until arrival to the refrigerator. On day 8, following an overnight fast, subjects visited the laboratory, delivered their first morning urine sample, blood samples were taken and body mass was measured as on day 1. Urine collection of each day was from 00:00 to 24:00. A reconstituted sample of 10 mL for each day consisted of samples from all samples that were collected during the 24 h period. The ratio of the volume of each urination per 24 h volume was calculated. The contribution of each urination to the reconstituted sample of 10 mL was calculated so that the volume ratio of each urination per 10 mL was the same to that of the volume ratio of each urination per the 24 h volume. Urine color was determined via the eight-point urine color chart developed by Armstrong (1994), urine and serum osmolality were measured in duplicate using freezing-point osmometer (Cryoscopic Osmometer, Osmomat 030, Gonotec). Urine and serum sodium and potassium were measured by ion selective electrode methods and urine creatinine was measured by the Jaffe enzymatic colorimetric method (Cobas Integra 400 plus). Urine specific gravity was measured with a pen refractometer (Master Reftractometer, Atago, cat. No. 2771). Urine volume was measured with an electronic digital scale (Soehnle Fiesta 65106). Hematocrit was determined via Micro Hematocrit Centrifuge (model, KHT-400), hemoglobin via spectrophotometer absorption (Pointe Scientific Inc. Hemoglobin Reagent Set, Canton, MI, USA). Finally, 7DD were analyzed with Diet Analysis plus version 6.1 (ESHA Research, Wadsworth Publishing Co. Inc., Salem, OR, USA) for the Greek population, PCN CESNID version 1.0 (Centre D’Ensenyament Superior De Nutricio I Dietetica, University of Barcelona, Barcelona, Spain) for the Spanish population and EBIS pro (German Food Database 3.1, University of Hohenheim, Stuttgart, Germany) for the German population.

Meteorological conditions (minimum and maximum temperature; relative humidity, precipitation) were provided by the nearest weather station of the center on each of the sampling days.

### 2.3. Statistical Analysis

Continuous variables are expressed as mean ± standard deviation for variables following normal distribution. Normal distribution of all continuous variables was tested with the parametric test Shapiro–Wilk or graphically assessed by histograms. Correlations between variables were evaluated using Pearson’s or Spearman’s correlation coefficient. Differences between genders and seasons (P1–P4) were derived through Student’s *t*-test for normally distributed variables. Differences among countries (P5) and among hyperhydrated, euhydrated, and dehydrated subjects were derived through One Way Anova test for normally distributed variables. *Post hoc* comparisons among countries were performed using Bonferroni test. The multivariate associations between variables were assessed using linear regression models, adjusted for all biologically plausible confounders. Subjects with missing some day value in one variable were not excluded from the analysis; the average of the week value was calculated from the remaining data. Statistical analysis was performed by SPSS package, version 16.1 (SPSS Inc., Chicago, IL, USA). We deemed statistical significance at α = 0.05.

## 3. Results

The population of the study that completed the protocol consisted of 573 subjects (age 39 ± 12 years; 280 females). 297 subjects (age 39 ± 12 years; 155 females) completed the protocol in the summer period. The mean BMI of males was 25.5 ± 4.2 kg/m^2^ and females 24.5 ± 4.9 kg/m^2^ (*p* = 0.012).

### 3.1. 24 h Urine Samples

Mean hydration indices (sodium, potassium, osmolality, urine volume, specific gravity, color) for 24 h urine samples from the seven days collection for males and females in winter and summer period and for each country are presented in Table 1.

Urine samples of men were more concentrated, as they had higher osmolality, specific gravity, and darker color. Women’s lower osmolality values are in agreement with the finding that quantities of sodium, potassium, and creatinine over a 24 h period (*p* < 0.001) are lower in women. There were also significant sex differences in summer period for most urine indices; females produced less concentrated urine of lower osmolality (*p* < 0.001) and excreted lower quantities of sodium and potassium (*p* < 0.001 and *p* = 0.016, respectively). Differences were observed in all urinary hydration indices (*p* < 0.001) among countries.

### 3.2. Blood Indices

Differences in serum osmolality (*p* = 0.001), hemoglobin and hematocrit were observed between genders (*p *< 0.001; Table 2). All indices were within the physiological ranges. In the summer population no differences were observed in serum glucose (*p* = 0.081), serum sodium (*p* = 0.166), and serum potassium (*p* = 0.092) between males and females.

### 3.3. Total Water Intake

Total water intake, water from beverages, water from foods, total energy intake, and energy from beverages are presented by gender, season, and country (Table 3). Water intake from beverages is correlated positively with total water intake (rho = 0.955, *p* < 0.001), energy intake (rho = 0.297, *p* < 0.001), and energy intake from beverages (rho = 0.576, *p* < 0.001). Daily water intake and water intake from beverages were higher in the summer compared to the winter period (*p* = 0.019 and *p* = 0.027 respectively). Differences were also observed between genders; when compared to females, males recorded higher total water (2.93 ± 1.10 L/day) and energy intake (2329 ± 686 kcal/day), consumed more water from beverages (2.27 ± 1.02 L/day) and received more calories from beverages (320 ± 219 kcal/day) (*p* < 0.001). Water intake derived from foods was higher in males compared to females totally (*p* = 0.027), but no differences were observed between seasons.

### 3.4. Classification of Subjects

**Figure 1:**
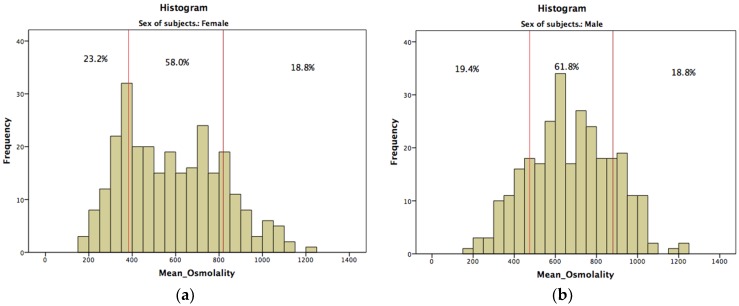
Distribution of hyperhydrated, euhydrated, and dehydrated (**a**) females and (**b**) males.

Subjects were further classified as hyperhydrated, euhydrated, and dehydrated according to reference values of 24 h urine osmolality for men and women [16,17]; classification is presented in summary in Figure 1, and in detail in Table 4. 

It was observed that 23.2%, 58.0%, and 18.8% of females and 19.4%, 61.8%, and 18.8% of males classified to the hyperhydrated, euhydrated, and dehydrated categories, respectively. Subjects that were classified to the hyperhydrated category also had higher total water intake (*p *< 0.001), greater urine volume (*p *< 0.001), lower specific gravity (*p *< 0.001), lighter color (*p *< 0.001), lower sodium and creatinine concentration (*p *< 0.001), and higher water intake from beverages (*p *< 0.001).

### 3.5. Linear Regression Model

Age (Beta = −4.033, *p *< 0.001), country (Beta = 81.196, *p *< 0.001), sex of subjects (Beta = 90.447, *p *< 0.001) and BMI (Beta = 9.146, *p *< 0.001) were significant predictors of 24 h urine osmolality while season and physical activity were not. The overall model fit was *R*^2^ = 0.208.

The age (Beta = 0.007, *p* = 0.009) and the country (Beta = –0.396, *p *< 0.01) of the subjects were predictors of 24 h urine volume. The overall model fit was *R*^2^ = 0.224. Country (Beta = −0.244, *p* = 0.001), sex of subjects (Beta = 0.473, *p *< 0.01), age (Beta = −0.013, *p* = 0.018), and BMI (Beta = 0.046, *p* = 0.002) were significant predictors of 24 h urine color. The overall model fit was *R*^2^ = 0.068.

## 4. Discussion

For the first time, a series of urine hydration indices from 24 h samples collected over seven consecutive days and blood hydration indices were measured in a sample of 573 healthy participants in three European countries and compared with water intake from seven day dietary records. The study contributes with new data to the literature referring to European hydration issues, allowing the observation of associations between water intake and hydration biomarkers.

Data in large populations groups of hydration indices are rare. Urine and blood hydration indices provide information that reflect water intake, water losses, and physiological processes. The present study is the first that measures fluid intake and urine output in a sample of the population from three countries at the same time using alike methodology. A demanding protocol for the collection of water intake information and urine samples, incorporates fluctuations in intake, and indices within the day and the week.

Urine hydration indices (24 h osmolality, volume, and color) were associated with age, gender, BMI, and country.

Country, gender, and age were found to be significant predictors of 24 h urine osmolality. This finding is in accordance with the review from Manz and Wentz [23], that describes large intercultural differences in 24 h urine osmolality values (from 360 to 860 mosm/kg). In our study values of 24 h urine osmolality, specific gravity, and color were significantly lower in the German population compared with the Spanish or Greek (*p* < 0.05 in all cases), while 24 h urine volume was significantly higher (*p *< 0.05). Total water intake and water intake from beverages was significantly higher in the German population than the Spanish or Greek populations. These differences may be attributed to dietary habits observed in the regions studied, partly related to the availability of local foods or beverages.

Regarding gender, women, in comparison to men had better hydration status. This may reflect different hydration, dietary and/or lifestyle choices between men and women. In general, women exhibit a more virtuous pattern of eating and food choices than men [24]. A study conducted in 23 countries showed that men’s dietary choices were less healthy, because health is less important motivation to them in the food domain [25]. Women seem to be more reflective about health issues and foods. Therefore, when it comes to adopting hydration guidelines there may be analogies with adopting nutritional guidelines in men and in women.

Age was a significant predictor for 24 h urine osmolality; as age increases urine osmolality decreases. This finding is in accordance with the study of Manz,* et al.* [26] where age related decrease in urine osmolality was observed. In the present study we found that country (Greece, Spain or Germany) was a significant predictor for 24 h urine volume. In a previous study conducted in adults (*n* = 10,079) large differences of mean 24-h urine volume identified between 52 centers all over the world [27]. It may be that lifestyle choices, environmental conditions, and other factors associated with living in different countries affect hydration status of the populations.

Using cutoffs for 24 h urine osmolality [16,17] approximately 60% of the subjects from three European countries were euhydrated. The distribution of hyperhydrated and dehydrated was similar for males and females. Hyperhydrated subjects consumed more fluids on daily basis (about 3.5 L/day), voided larger volumes (2.5 L/day) and provided urine samples that were less concentrated.

It must be noted that results presented herein derive from a volunteer sample of subjects from three countries and may not be generalizable to the entire European population. Further analysis of data by country of origin, age group, physical activity level, *etc*., may reveal the influence of these factors to the hydration status of the studied group.

In our study the mean total water intake was 2.75 ± 1.01 L/day. Previous studies [23,28] have reported daily fluid intake using Food Frequency Questionnaires (FFQ) or 24 h recall. These tools may underestimate water intake [29]. Gibson and Shirreffs [30] found that total water intake from foods and beverages was 2270 g/day in UK population and observed fluctuations in water intake during a week, recording higher consumption of drinks on Fridays and Saturdays. A seven-day fluid specific record given in 13 different countries found that mean daily water intake was 1980 mL/day, with highest fluid intake recorded in Germany (2.47 L/day) and the lowest in Japan (1.50 L/day) [31]. Water intake guidelines are frequently complex and not always harmonized. For example, D-A-CH suggests the intake of water from 1 mL/kcal of energy intake for adults [32], European Food Safety Authority (EFSA) suggests 2.5 and 2.0 L/day from males and females [4], and I.O.M. suggests 3.7 and 2.7 L/day for males and females respectively [5]. Recommendations for the Europeans are lower than US population and refer to water intake from all sources.

The contribution of foods in water intake was 24% (approximately 700 mL) with no differences reported between genders. This finding is similar to those provided in the scientific opinion of EFSA [4] and in previous studies [3,33].

The daily intake of beverages (2.1 ± 0.91 L/day) contributes approximately 290 kcal (13% of energy intake). Data of total energy intake are in accordance with previous published studies. In particular, total energy intake in the EPIC study was 2508 (2167, 2950) kcal for men and 1999 (1741, 2348) kcal for women and in the ATTICA study was 2595 ± 877 kcal for men and 2132 ± 658 kcal for women [34,35]. In the present study 24 h total water intake is strong and positively correlated with 24 h water intake from beverages (*r* = 0.955, *p *< 0.001) and energy intake from beverages (*r* = 0.543, *p* < 0.001). Moreover, differences were observed in the total water intake between seasons (*p* = 0.019). This difference could be explained due to high temperatures in summer period compared to winter, which reflects higher fluid intake and sweat loss. However, this difference of 200 mL/day between seasons is lower than a previous study in Greece [33]. This difference could be explained due to different environmental conditions (temperature, humidity) and alternative lifestyle choices of the participants [20].

## 5. Conclusions

In conclusion, in a free-living population from German, Spain, and Greece approximately 60% were euhydrated while approximately 20% were hyperhydrated and 20% dehydrated on average over a seven-day period. Differences observed on urine and blood hydration indices, total water intake, and water intake from beverages and foods suggest that a variety of dietary or lifestyle factors that may be associated with improving hydration status.

## Figures and Tables

**Table 1:** 24 h urine hydration indices of participants in winter and summer.

		Sodium (mEq/Day)	Potassium (mEq/Day)	Creatinine (mg/Day)	Urine Osmolality (mOsmol/kg·Η_2_Ο)	Urine Volume (L)	USG	Color
Winter	Male	178.4 ± 51.5	76.0 ± 20.1	1738.4 ± 523.0	652 ± 211	1.66 ± 0.62	1.018 ± 0.005	4.4 ± 1.4
	Female	162.9 ± 56.7	68.8 ± 21.8	1335.6 ± 404.1	571 ± 197	1.70 ± 0.72	1.016 ± 0.005	4.1 ± 1.3
	Total	171.4 ± 54.4	72.7 ± 21.2	1555.2 ± 512.8	615 ± 209	1.68 ± 0.66	1.017 ± 0.005	4.2 ± 1.4
Summer	Male	181.9 ± 50.1	76.2 ± 25.7	1820.6 ± 451.6	698 ± 192	1.61 ± 0.70	1.018 ± 0.005	4.6 ± 1.2
	Female	145.6 ± 53.1	68.5 ± 28.3	1290.0 ± 474.4	596 ± 251	1.63 ± 0.77	1.015 ± 0.006	3.9 ± 1.6
	Total	162.8 ±54.7	72.2 ± 27.3	1543.7 ± 533.6	645 ± 230	1.62 ± 0.73	1.017 ± 0.006	4.2 ± 1.5
	P1	0.021	0.005	<0.001	0.001	0.586	0.003	0.069
	P2	<0.001	0.016	<0.001	<0.001	0.789	<0.001	<0.001
	P3	0.065	0.789	0.795	0.111	0.370	0.679	0.983
Winter & Summer	Total Male	180.1 ± 50.8	76.1± 22.9	1779.1 ± 489.9	675 ± 203	1.63 ± 0.66	1.018 ± 0.005	4.5 ± 1.3
	Total Female	153.2 ± 55.3	68.6 ± 25.6	1310 ± 444.7	585 ± 229	1.66 ± 0.74	1.015 ± 0.006	4.0 ± 1.5
	Total Sample	166.9 ± 54.7	72.4 ± 24.6	1549.1 ± 523.4	631 ± 221	1.65 ± 0.70	1.017 ± 0.005	4.2 ± 1.4
	P4	<0.001	<0.001	<0.001	<0.001	0.619	<0.001	<0.001
Country	German	162.2 ± 50.3 *^,#^	77.9 ± 24.1 ^#^	1454.0 ± 401.0 *	492 ± 170 *^,#^	2.13 ± 0.76 *^,#^	1.014 ± 0.005 *^,#^	4.4 ± 1.3 ^#^
	Spain	192.8 ± 51.7 ^+^	74.0 ± 27.8 ^+^	1807.9 ± 621.2 ^+^	753 ± 180^+^	1.40 ± 0.49	1.019 ± 0.004 ^+^	4.4 ± 1.5 ^+^
	Greece	143.8 ± 51.0	64.4 ± 18.7	1377.9 ± 415.3	658 ± 224	1.36 ± 0.50	1.017 ± 0.006	4.0 ±1.5
	P5	<0.001	<0.001	<0.001	<0.001	<0.001	<0.001	0.008

*p*-values derived through Student’s *t*-test for differences between genders and season; and one-way ANOVA among countries; * significant difference between German and Spain; # significant difference between German and Greece; ^+^ significant difference between Spain and Greece; P1 refers to comparisons between gender for winter, P2 refers to comparisons between gender for summer, P3 refers to comparisons between summer and winter for the total sample (males and females together); P4 refers to comparisons between males and females (winter and summer together); P5 refers to comparisons between countries.

**Table 2:** Blood and serum hydration indices of participants in winter and summer.

		Hb (g/dL)	Htc (%)	Glucose (mmol/L)	Serum Osmolality (mOsmol/kg Η_2_Ο)	Sodium (mEq/L)	Potassium (mEq/L)
Winter	Male	15.3 ± 1.5	45 ± 3	4.67 ± 0.46	297 ± 10	143.0 ± 4.9	4.4 ± 0.4
	Female	14.1 ± 1.6	42 ± 4	4.73 ± 0.52	294 ± 10	141.6 ± 3.9	4.4 ± 0.4
	Total	14.7 ± 1.7	43 ± 4	4.70 ± 0.49	296 ± 10	142.4 ± 4.5	4.4 ± 0.4
Summer	Male	15.5 ± 1.5	45 ± 3	5.02 ± 1.09	293 ± 7	143.3 ± 5.1	4.5 ± 0.5
	Female	14.0 ± 1.6	41 ± 4	4.88 ± 1.59	291 ± 8	144.7 ± 11.3	4.6 ± 0.6
	Total	14.7 ± 1.7	43 ± 4	4.94 ± 1.37	292 ± 8	144.0 ± 8.9	4.6 ± 0.6
	P1	<0.001	<0.001	0.339	0.015	0.020	0.580
	P2	<0.001	<0.001	0.388	0.081	0.166	0.064
	P3	0.824	0.397	0.005	<0.001	0.005	<0.001
Winter & Summer	Total Male	15.4 ± 1.5	45 ± 3	4.84 ± 0.85	295 ± 9	143.1 ± 5.0	4.5 ± 0.5
	Total Female	14.0 ± 1.6	42 ± 4	4.81± 1.24	292 ± 9	143.3 ± 8.9	4.5 ± 0.5
	Total Sample	14.7 ± 1.7	43 ± 4	4.83 ± 1.06	294 ± 9	143.2 ± 7.2	4.5 ± 0.5
	P4	<0.001	<0.001	0.724	0.001	0.717	0.159
Country	German	14.3 ± 1.3 *	43 ± 3 *^,#^	4.52 ± 1.43 *^,#^	298 ± 11^*#^	141.0 ± 1.7 ^#^	4.5 ± 0.4 ^#^
	Spain	15.2 ± 1.3^+^	46 ± 3 ^+^	4.96 ± 0.39	289 ± 8 ^+^	141.2 ± 2.6 ^+^	4.4 ± 0.5 ^+^
	Greece	14.6 ± 2.3	41 ± 4	5.07 ± 0.97	294 ± 6	148.5 ± 11.4	4.6 ± 0.6
	P5	<0.001	<0.001	<0.001	<0.001	<0.001	<0.001

*p*-values derived through Student’s *t*-test for differences between genders and season; and one-way ANOVA among countries; * significant difference between German and Spain; ^#^ significant difference between German and Greece; ^+^ significant difference between Spain and Greece; P1 refers to comparisons between gender for winter, P2 refers to comparisons between gender for summer, P3 refers to comparisons between summer and winter for the total sample (males and females together), P4 refers to comparisons between males and females (winter and summer together), and P5 refers to comparisons between countries.

**Table 3:** Daily intake of water from all sources, from beverages and foods, separately of participants in winter and summer periods.

		Total Water Intake (L/Day)	Water from Beverages (L/Day)	Water from Foods (L/Day)	Total Energy Intake (kcal/Day)	Energy from Beverages (kcal/Day)
Winter	Male	2.77 ± 1.10	2.12 ± 1.09	0.67 ± 0.31	2248 ± 659	302 ± 203
	Female	2.49 ± 0.80	1.89 ± 0.71	0.61 ± 0.25	1913 ± 477	258 ± 143
	Total	2.64 ± 0.98	2.01 ± 0.94	0.64 ± 0.29	2093 ± 605	282 ± 179
Summer	Male	3.09 ± 1.07	2.41 ± 0.93	0.69 ± 0.29	2413 ± 706	338 ± 233
	Female	2.61 ± 0.91	1.97 ± 0.75	0.64 ± 0.29	1989 ± 580	254 ± 141
	Total	2.84 ± 1.02	2.18 ± 0.87	0.68 ± 0.29	2192 ± 676	294 ± 195
	P1	0.014	0.034	0.075	<0.001	0.038
	P2	<0.001	<0.001	0.152	<0.001	0.001
	P3	0.019	0.027	0.339	0.068	0.430
Winter & Summer	Total Male	2.93 ± 1.10	2.27 ± 1.02	0.68 ± 0.30	2329 ± 686	320 ± 219
	Total Female	2.55 ± 0.86	1.93 ± 0.73	0.63 ± 0.27	1955 ± 537	256 ± 142
	Total Sample	2.75 ± 1.01	2.10 ± 0.91	0.66 ± 0.29	2148 ± 644	288 ± 188
	P4	<0.001	<0.001	0.027	<0.001	<0.001
Country	German	3.29 ± 0.98 *^,#^	2.49 ± 0.87 *^,#^	0.81 ± 0.27 *^,#^	2412 ± 609 *^,#^	358 ± 240 *^,#^
	Spain	2.55 ± 0.98	1.96 ± 0.95	0.61 ± 0.29 ^+^	2214 ± 633 ^+^	296 ± 145 ^+^
	Greece	2.35 ± 0.77	1.82 ± 0.74	0.54 ± 0.23	1777 ± 512	203 ± 113
	P5	<0.001	<0.001	<0.001	<0.001	<0.001

*p*-values derived through Student’s *t*-test for differences between genders and season; and one-way ANOVA among countries; * significant difference between German and Spain; ^#^ significant difference between German and Greece; ^+^ significant difference between Spain and Greece; P1 refers to comparisons between genders for winter, P2 refers to comparisons between gender for summer, P3 refers to comparisons between summer and winter for the total sample (males and females together), P4 refers to comparisons between males and females (winter and summer together), and P5 refers to comparisons between countries.

**Table 4:** Water intake and 24 h urine indices of females and males, according to the categories of hydration status based to urine osmolality.

Categories of Hydration Status According to Urine Osmolality (mOsm/kg H_2_0)				
	Hyperhydrated	Euhydrated	Dehydrated	*p*
	(<383)	(383 to 810)	(>810)	
*Females, % (n)*	23.2 (64)	58.0 (160)	18.8 (52)	
Total water intake (L/day)	3.36 ± 1.02	2.42 ± 0.61	2.02 ± 0.65	<0.001
Water from beverages (L/day)	2.60 ± 0.91	1.81 ± 0.49	1.53 ± 0.57	<0.001
24 h urine volume (L)	2.51 ± 0.73	1.54 ± 0.52	1.00 ± 0.25	<0.001
24 h urine specific gravity	1.009 ± 0.002	1.016 ± 0.004	1.023 ± 0.003	<0.001
24 h urine color	3.0 ± 1.2	3.9 ± 1.2	5.5 ± 1.2	<0.001
24 h urine Na (mEq/day)	129.4 ± 37.1	158.7 ± 59.1	166.3 ± 54.2	<0.001
24 h urine K (mEq/day)	73.9 ± 36.5	67.6 ± 21.6	65.4 ± 20.1	0.153
24 h urine creatinine (mg/day)	1137.6 ± 249.1	1362.5 ± 494.0	1363.6 ± 429.6	0.002
	(<475)	(475 to 880)	(>880)	
*Males, % (n)*	19.4 (55)	61.8 (181)	18.8 (54)	
Total water intake (L/day)	3.59 ± 1.04	2.8 ± 0.99	2.64 ± 1.25	<0.001
Water from beverages (L/day)	2.83 ± 1.00	2.15 ± 0.86	2.08 ± 1.31	<0.001
24 h urine volume (L)	2.45 ± 0.69	1.56 ± 0.46	1.00 ±0.24	<0.001
24 h urine specific gravity	1.011 ± 0.002	1.018 ± 0.003	1.025 ± 0.02	<0.001
24 h urine color	3.6 ± 1.4	4.3 ± 1.1	5.9 ± 1.0	<0.001
24 h urine Na (mEq/day)	156.4 ± 50.0	187.7 ± 46.4	180.1 ± 59.5	<0.001
24 h urine K (mEq/day)	76.9 ± 19.9	77.7 ± 24.4	69.8 ± 20.2	0.091
24 h urine creatinine (mg/day)	1517.7 ± 399.02	1862.9 ± 483.3	1771.2 ± 522.1	<0.001

Results are presented as mean ± SD; *p*-values derived through one-way ANOVA for the normally distributed variables.
